# Brain cholinergic terminal density utilizing [^18^F]‐fluoroethoxybenzovesamicol PET in adults with Down's syndrome: Relationship to amyloid PET and cognitive performance

**DOI:** 10.1002/alz.70134

**Published:** 2025-04-06

**Authors:** Jason K. Russell, Alexander C. Conley, Brian D. Boyd, John Patrick Begnoche, Rachel Schlossberg, Allison Stranick, Adam J. Rosenberg, Lealani Mae Y. Acosta, Dann Martin, Yasmeen Neal, Michael S. Rafii, Julie Dumas, Paul A. Newhouse

**Affiliations:** ^1^ Center for Cognitive Medicine Department of Psychiatry and Behavioral Sciences Vanderbilt University Medical Center Nashville Tennessee USA; ^2^ Vanderbilt University Institute of Imaging Science Vanderbilt University Medical Center Nashville Tennessee USA; ^3^ Department of Radiology and Radiological Sciences Vanderbilt University Medical Center Nashville Tennessee USA; ^4^ Department of Neurology Vanderbilt University Medical Center Nashville Tennessee USA; ^5^ Department of Clinical Radiology and Radiological Sciences Vanderbilt University Medical Center Nashville Tennessee USA; ^6^ Alzheimer's Therapeutic Research Institute Keck School of Medicine University of Southern California San Diego California USA; ^7^ Department of Psychiatry University of Vermont Burlington Vermont USA; ^8^ Geriatric Research Education, and Clinical Center Veterans Affairs Tennessee Valley Health System Nashville Tennessee USA

**Keywords:** [^18^F]‐FEOBV, acetylcholine, amyloid, cholinergic, cognition, Down syndrome, PET imaging

## Abstract

**BACKGROUND:**

Adults with Down syndrome (DS) have increased risk of Alzheimer's disease (AD). The cholinergic system declines in AD, underlying many cognitive deficits. We investigated the relationship between amyloid accumulation and cholinergic terminal density in adults with DS compared to amyloid‐matched controls.

**METHODS:**

A total of 15 non‐demented adults with DS and 15 amyloid‐matched healthy controls were assessed for [^18^F]‐FEOBV uptake differences and [^18^F]‐FEOBV uptake relationships with amyloid accumulation and cognitive performance.

**RESULTS:**

Adults with DS displayed greater [^18^F]‐FEOBV uptake than controls, with a similar uptake pattern. Amyloid‐associated differences in [^18^F]‐FEOBV uptake were observed in adults with DS. [^18^F]‐FEOBV uptake in adults with DS was positively associated with cognition.

**DISCUSSION:**

Adults with DS display higher [^18^F]‐FEOBV uptake than amyloid‐matched controls but relatively lower [^18^F]‐FEOBV uptake in individuals with elevated amyloid. Thus, the cholinergic system appears to be adversely affected by AD pathology in individuals with DS, which may be relevant to cognitive decline.

**Highlights:**

- Adults with DS display greater cholinergic terminal density in specific ROIs than amyloid‐match controls.
- Adults with DS exhibit a similar pattern of cholinergic terminal density across the brain.
- The first association of cholinergic terminal density with AD pathology in non‐demented adults with DS.
- Adults with DS display a greater cholinergic terminal decline in association with amyloid accumulation than neurotypically developed age‐matched controls.
- Region‐specific cholinergic terminal density associated with cognitive performance in adults with DS.

## 1 BACKGROUND

Individuals with Down syndrome (DS) display triplication of the amyloid precursor protein (APP) gene, leading to early and widespread amyloid accumulation and a greatly increased risk of developing Alzheimer's disease (AD) compared to the general population.^1^, ^2^, ^3^ While medical advancements have significantly increased the life expectancy for individuals with DS,^4^ it is reported that 80% of adults with DS will develop symptomatic AD by 65 years of age.^5^ Thus, given the increased life expectancy in individuals with DS, AD is now the primary cause of death in individuals with DS.^6^

The central cholinergic system is known to decline due to the development of AD and is believed to underlie many of the observed cognitive and behavioral deficits observed in this disease.^7^, ^8^, ^9^ The central cholinergic system has been demonstrated to be one of the first neurotransmitter systems to be affected during the development of AD, with central cholinergic degeneration observed in individuals with subjective cognitive decline and mild cognitive impairment.^10^, ^11^ Numerous studies have linked specific AD‐related pathology to declining cholinergic integrity. Increased amyloid burden has been associated with reduced cholinergic integrity measured through basal forebrain volumetry and basal forebrain tractography.^12^, ^13^, ^14^ At the same time, other clinical and preclinical studies have linked tau accumulation to cholinergic pathology.^15^, ^16^ In adults with DS who were both non‐demented and demented, cholinergic basal forebrain atrophy was observed to be associated with both amyloid and tau biomarkers, and cholinergic basal forebrain degeneration in adults with DS was associated with impaired cognitive performance.^17^

[^18^F]‐Fluoroethoxybenzovesamicol ([^18^F]‐FEOBV) is a vesamicol derivative that binds the vesicular acetylcholine transporter (VAChT) uniquely expressed presynaptically in cholinergic nerves,^18^ thus labeling presynaptic cholinergic nerve terminals. [^18^F]‐FEOBV has been utilized to study cholinergic terminal density in healthy aging^19^, ^20^, ^21^ and AD research.^9^, ^22^, ^23^, ^24^ [^18^F]‐FEOBV uptake has been observed to be reduced in older age,^19^, ^20^ and individuals with AD display an accelerated decline in [^18^F]‐FEOBV uptake with increased disease progression.^24^ Lower [^18^F]‐FEOBV uptake is associated with worse cognitive performance.^9^

We have recently demonstrated that non‐demented adults with DS display increased [^18^F]‐FEOBV uptake in a range of cortical and subcortical regions compared to neurotypically developed age‐matched controls and a greater age‐related reduction in [^18^F]‐FEOBV uptake than is observed in the neurotypical population.^25^ Here, we investigate whether observed age‐related differences in [^18^F]‐FEOBV binding are associated with positron emission tomography (PET) measures of AD pathology. Previous studies have investigated changes in cholinergic basal forebrain volume associated with AD fluid‐based biomarkers in adults with DS at different stages of dementia.^17^ The current study is the first to assess how changes in cholinergic terminal density before the development of dementia in adults with DS are associated with developing AD‐related pathology in a population at high risk for the development of dementia due to AD. In addition, this study utilizes [^18^F]‐FEOBV PET imaging, which provides detailed information about which anatomical regions display cholinergic terminal density differences, in contrast to basal forebrain volumetry. Furthermore, this study investigated the relationship between [^18^F]‐FEOBV uptake and cognitive performance in non‐demented adults with DS. These data, in a population with genetically determined AD, provide a unique insight into the relationship between cholinergic terminal density and amyloid pathology during AD development.

We hypothesized that [^18^F]‐FEOBV uptake would be greater in a wide range of cortical and subcortical areas in adults with DS than amyloid‐matched controls. Further, we perform exploratory analyses assessing the relationship between amyloid accumulation, as measured by amyloid PET, and [^18^F]‐FEOBV uptake. Specifically, we hypothesized that increased amyloid accumulation measured in centiloids would be negatively associated with regional [^18^F]‐FEOBV uptake. We also performed exploratory analysis assessing whether there was an interaction between group (adults with DS and amyloid‐matched neurotypically developed controls) and amyloid accumulation on [^18^F]‐FEOBV uptake. Given the relationship between cholinergic integrity and cognition described in neurotypical individuals, we hypothesize that [^18^F]‐FEOBV uptake would be positively associated with cognitive performance, specifically memory and executive function‐based tasks, in non‐demented adults with DS.

## 2 METHODS

### 2.1 Participants

A total of 15 non‐demented adults with DS, with an average age of 36.3 years old (range 23–50 years old, 7 male, 8 female), were recruited at Vanderbilt University Medical Center from the Trial Ready Cohort—Down Syndrome (TRC‐DS) study.^26^ In this cohort study, participants undergo amyloid and tau PET imaging, magnetic resonance imaging (MRI) imaging, and cognitive assessments (NCT04165109). In addition to study procedures for TRC‐DS, participants at Vanderbilt University Medical Center underwent an [^18^F]‐FEOBV PET scan as part of an approved substudy (NCT05231798). The Vanderbilt University Medical Center Institutional Review Board approved both the TRC‐DS study and the cholinergic substudy, and participants or legally authorized representatives gave written informed consent in accordance with the Declaration of Helsinki.

Fifteen neurotypically developed adults (61.5 years old, range 53–69; 15 females) from a separate study (Cognitive Health after Menopause [CHAMP]; NCT04129060) were selected based on matching global amyloid accumulation measured in centiloids. Individuals were cognitively normal and had no diagnosis of dementia or other neurologic conditions. Cognitive status was established through a detailed neuropsychiatric assessment including the Montreal cognitive assessment (MoCA),^27^ Beck Anxiety Index,^28^ Beck Depression Index,^29^ Everyday Cognition participant and informant questionnaires,^30^ Structured Clinical Interview for DSM‐4 (SCID),^31^ Brief Cognitive Rating Scale,^32^ Global Deterioration Scale,^33^ Mattis Dementia Rating Scale‐2 (DRS‐2),^34^ Delis‐Kaplan Executive Function System (D‐KEFS) Trails & Verbal Fluency,^35^ and Repeatable Battery for the Assessment of Neuropsych Status (RBANS).^36^ For this study, participants were recruited at Vanderbilt University Medical Center and the University of Vermont and underwent MRI, amyloid PET, and [^18^F]‐FEOBV PET scans. Amyloid matched participants all underwent MRI and PET scans at Vanderbilt University Medical Center. The CHAMP study was a multicenter study approved by the University of Vermont Institutional Review Board, with the [^18^F]‐FEOBV PET imaging substudy approved by the Vanderbilt University Medical Center Institutional Review Board. All participants or their legally authorized representatives for TRC‐DS, CHAMP, and the associated PET substudies gave written informed consent in accordance with the Declaration of Helsinki. Study data was collected and managed using reserch electronic data capture (REDCap) tools hosted at Vanderbilt University Medical Center.^37^, ^38^ For full participant demographics, see Table 1; for amyloid matching, see Table S1.

**RESEARCH IN CONTEXT**

1. **Systematic review**: The central cholinergic system declines in Alzheimer's disease (AD) and underlies many of the observed cognitive deficits. However, few studies have investigated the relationship between AD pathology and cholinergic integrity in adults with Down syndrome (DS) prior to the onset of dementia.
2. **Interpretation**: Our findings indicate the pattern of cholinergic terminal density across the brain is similar between adults with DS and neurotypical controls, however adults with DS display elevated cholinergic terminal density in select regions. Exploratory data suggests lower cholinergic terminal density is observed with the development of early amyloid pathology in adults with DS, in contrast to amyloid‐matched neurotypical controls.
3. **Future directions**: These data suggest the central cholinergic system is more sensitive to AD pathology in adults with DS than neurotypically developed controls. Ongoing studies will follow participants longitudinally to confirm this hypothesis and identify potential mechanisms for this increased sensitivity to AD‐related pathology.

**TABLE 1 alz70134-tbl-0001:** Participant demographics.

Parameter	Adults with Down syndrome	Neurotypically developed adults
*N*	15	15
Age (range)	36.3 (23–50)	61.5 (53–69)
Sex (Female %)	53.3	100
Centiloid (range)	9.8 (–3.9–63.6)	9.1 (–4.7–61.9)
Race (%)		
White	93.3	86.7
Black or African American	0	13.3
Mixed	6.7	0
KBIT‐2: IQ (range)	48.9 (40–70)	NA
DSMSE: total score (range)	75.5 (49–94)	NA
mCRT: free recall (range)	21.5 (12–31)	NA
TOPF: IQ estimate (range)	NA	121.5 (105–131)
MoCA (range)	NA	27.6 (25–30)

Abbreviations: DSMSE, Down Syndrome Mental State Examination; KBIT‐2, Kaufman Brief Intelligence Test—2nd edition; mCRT, modified Cued Recall Task; MoCA, Montreal Cognitive Assessment; TOPF, Test of Premorbid Functioning.

### 2.2 DS participant cognitive assessment

Participants from the DS cohort underwent the Kaufman Brief Intelligence Test—2nd edition (KBIT‐2) to establish premorbid intellectual disability. The modified Cued Recall Task (mCRT),^39^ the Down Syndrome Mental State Examination (DSMSE),^40^ and the Stroop Dog and Cat Task^41^ to assess cognitive performance. All three tasks have previously been validated in participants with DS.^39^, ^40^, ^41^ mCRT is a task adapted to measure free and cued recall in individuals with intellectual disability. For associations with cholinergic terminal density, the total immediate free recall was used, as this has previously been demonstrated to be sensitive to measures of cholinergic integrity in adults with DS.^17^ The commonly used measure of total recall (cued and free) was not utilized as most participants were at ceiling with this measure. The DSMSE is a battery of cognitive assessments testing a range of domains, including apraxia, language, visuospatial, and object and location memory. The relationship between total DSMSE score and [^18^F]‐FEOBV uptake was assessed with additional analysis to investigate relationships with the memory and non‐memory sub‐scores. The Stroop Dog and Cat task is a modified Stroop task of executive functioning. The measure used is a composite of the Cat Dog Switch Time Score and the Cat Dog Switch Error Score. The switch time score is the time taken to complete the switch trial minus the time to complete the initial trial, and the switch error score is the number of errors on the switch trial minus the number of errors on the initial trail. Both scores were z‐transformed and combined into an unweighted composite score using the equation 0.5 x switch time z‐score + 0.5 x switch error z‐score.[Table alz70134-tbl-0001]

### 2.3 MRI imaging acquisition

For the participants with DS, MRI scans were performed at Vanderbilt University Medical Center on a research‐dedicated Philips 3.0T Ingenia Elition X (Philips Medical Systems, Best, the Netherlands). For PET imaging registration, T1‐weighted scans with TR = 6.7 ms, TE = 3.1 ms, and a spatial resolution of 1 × 1 × 1.2 mm^3^ (170 slices and 334‐s duration) were utilized.

For the neurotypical individuals, MRI scans were performed on a research‐dedicated Philips 3.0T Ingenia CX (Philips Medical Systems, Best, the Netherlands) at Vanderbilt University Medical Center. For PET image registration, T1‐weighted scans with TR = 6.3 ms, TE = 2.9 ms, and a spatial resolution of 1 × 1 × 1 mm^3^ (225 slices and a 338‐second duration) were utilized.

### 2.4 [^18^F]‐FEOBV synthesis

[^18^F]‐FEOBV was prepared similar to the published method.^42^ Briefly, ^18^F^−^ was made by irradiating enriched [^18^O]‐water with protons. The ^18^F^−^ was separated from the [^18^O]‐water by trapping the ^18^F^−^ on an anion exchange cartridge. The synthesis of [^18^F]‐FEOBV was divided into three steps: (1) labeling, (2) purification, and (3) reformulation. Labeling was carried out using ^18^F/K_2_CO_3_/Kryptofix complex in dimethylsulfoxide (DMSO). The labeled intermediate was diluted with the high‐performance liquid chromatography (HPLC) mobile phase and injected onto the HPLC. The desired radioactive peak was isolated and diluted with sterile water (50 mL) followed by transfer on a C18 Sep‐Pak Plus. The Sep‐Pak was then washed with sterile water for injection (10 mL) and subsequently eluted with ethanol (0.5 mL), followed by saline (9.5 mL). This mixture was then passed through a 0.22 µm sterilizing filter and into the final vial. Samples were then removed for analysis of product quality. After final purification, typical yields were in the range of 2%–10% (non‐decay corrected). The overall synthesis time was 75 min, specific activity at the end of synthesis was 15.3 Ci/µmol (range 5 Ci/µmol—41.5 Ci/µmol).

### 2.5 PET imaging acquisition

All 30 participants underwent amyloid ([^11^C]‐Pittsburgh compound B ([^11^C]‐PiB) for the DS participants and [^18^F]‐Florbetapir for the neurotypically developed individuals) and cholinergic ([^18^F]‐FEOBV) PET. [^11^C]‐PiB and [^18^F]‐FEOBV were synthesized at the Vanderbilt University Medical Center Radiochemistry Core, [^18^F]‐Florbetapir was synthesized by Avid Radiopharmaceuticals.

For [^18^F]‐FEOBV acquisition, participants received 6.5mCi ± 10% via a slow I.V. bolus followed by a saline flush. Following a 3‐h uptake, data acquisition was initiated. Scans lasted 30 min, with each scan consisting of six 300‐s frames. For [^11^C]‐PiB acquisition, participants received 15mCi ± 10% via a slow I.V. bolus followed by a saline flush. Following a 50‐min uptake, data acquisition was initiated. Scans lasted 20 min, with each scan consisting of four 300‐s frames. For [^18^F]‐Florbetabir acquisition, participants received 10mCi ± 10% via a slow I.V. bolus followed by a saline flush. Following a 50‐min uptake, data acquisition was initiated. Scans lasted 20 min, with each scan consisting of four 300‐s frames. Only the first two frames were utilized for calculating centiloid values in the participants receiving [^18^F]‐Florbetabir.^43^ All PET scans had a voxel size of 2 mm isotropic and a field of view (FOV) of 256 mm and were performed using a Philips Vereos digital positron emission tomography‐computed tomography (PET/CT) system at Vanderbilt University Medical Center.

### 2.6 PET imaging data processing

[^18^F]‐FEOBV PET imaging analysis was performed using a voxel‐based approach. Initially, images were motion‐corrected using Motion Correction by Functional Magnetic Resonance Imaging of the Brain's (FMRIB's) Linear Image Registration Tool (MCFLIRT) in FMRIB Sortware Library (FSL). PetSurfer (https://surfer.nmr.mgh.harvard.edu/fswiki/PetSurfer)^44^, ^45^ was used to co‐register the T1 MRI and [^18^F]‐FEOBV PET images and apply a partial volume correction to the PET scans using the region‐based voxel‐wise (RBV) method.^46^ [^18^F]‐FEOBV uptake was then referenced to the eroded supraventricular white matter in a whole‐brain voxel‐wise manner.^20^ [^18^F]‐FEOBV PET images were transformed to MNI‐152 space using Advanced Normalization Tools for Python (ANTsPy) and ANTsPyNet^47^ in Python v3.12.14. First, the coregistered T1‐weighted MRI scans had brain extraction performed using ANTsPyNet and were transformed to Montreal Neurological Institute (MNI)‐152 space using symmetric normalization. The [^18^F]‐FEOBV PET images were transformed to MNI‐152 space using the *fwdtransforms* function. As the cerebellar cholinergic system is reported to be unaffected by AD‐related pathology,^24^ we focused our analyses on cortical and subcortical grey matter regions to limit unnecessary voxel‐wise comparisons. To remove white matter and cerebellar regions from the analysis study‐specific masks were generated using Nilearn (v0.10.4)^48^ and ANTsPyNet in Python (v3.12.4). T1‐weighted MRI images in MNI‐152 space were used to calculate individualized grey matter masks in Nilearn, and a whole cerebellum mask with the deep atropos tool in ANTsPyNet. The cerebellar mask was inverted using Nilearn to remove it from the analysis and combined with the grey matter mask. The individualized masks were then combined in a probabilistic manner with a threshold of 0.3 in Nilearn to generate a study‐specific mask. To further reduce unnecessary comparisons, once the [^18^F]‐FEOBV PET images were transformed to MNI‐space, they were averaged, and only voxels that had an SUVR‐value of > 1 were included in the voxel‐based analysis. The analyses assessing the relationship between cognitive performance and [^18^F]‐FEOBV uptake were also performed following transformation to a DS‐specific template. The template used was the cognitively stable—DS template described by Queder et al.^49^ The methods for spatial transformation to the DS‐specific template were the same as described for the MNI template. Cerebellar and grey matter masks were generated in MNI‐152 space and inverse transformed using the previously generated transformation matrices via the *invtransforms* function to subject space and then forward transformed via the *fwdtransforms* function to the DS‐specific template.

[^11^C]‐PiB and [^18^F]‐Florbetabir PET images were analyzed utilizing the standardized centiloid approach to assess the effects of global amyloid deposition.^43^, ^50^ For centiloid analysis, the PMOD NEURO tool (PMOD Technologies LLC, Switzerland) was utilized. The centiloid analysis was validated for both amyloid radiotracers using the Global Alzheimer's Association Interactive Network (GAAIN) centiloid project datasets as previously described.^50^

### 2.7 Region of interest‐based analysis

Region of interest (ROI) ‐based analyses were performed in subject space using FreeSurfer and PetSurfer. PET images were motion‐corrected using MCFLIRT in FSL prior to coregistration to the T1‐weighted MRI in PetSurfer. FreeSurfer (http://surfer.nmr.mgh.harvard.edu/) was used for cortical reconstruction and volumetric segmentation of the T1‐weighted MRIs. The parcellations generated were used to calculate the standard uptake value ratios (SUVRs) normalized to the eroded supratentorial white matter.

### 2.8 Voxel‐based analysis and statistics

Voxel‐wise general linear models (GLMs) assessing the relationship between [^18^F]‐FEOBV uptake and global centiloid were performed in Nilearn. For this, centiloid was the variable of interest with age included as a covariate. Due to the close association between age and amyloid accumulation in adults with DS and the large age difference between the DS and amyloid‐matched control group, results are reported with and without age as a covariate. To investigate the interaction of group (DS or neurotypically developed) and amyloid accumulation (in centiloids), a GLM assessing the interaction between group and centiloid value was performed. A whole‐brain assessment of centiloid‐associated change in the two groups was performed to inform the directionality of any interaction effects. The beta‐value for the association between centiloid and [^18^F]‐FEOBV uptake was masked to include only negative associations. To assess the relationship between [^18^F]‐FEOBV uptake and cognitive performance, whole‐brain voxel‐wise GLMs were performed, with KBIT‐2 score included as a covariate to account for premorbid intellectual disability. Due to relatively small group sizes and anticipated small effect sizes due to the adults with DS being non‐demented, a relatively liberal *p*‐value of *p* < 0.005 was used with a minimum cluster size of 50 to reduce the likelihood of spurious findings. Results are visualized using the xjView toolbox (https://www.alivelearn.net/xjview). Figure S1 comparing age and amyloid accumulation was generated in R (version 4.4.1) with Locally Estimated Scatterplot Smoothing (LOESS) with a span of 1 used to generate the curve. For ROI‐based analyses, group‐wise differences were determined using a student's t‐test. Rank ordering of [^18^F]‐FEOBV SUVRs across ROIs between groups was compared using Kendall's Tau rank correlation. GLMs were used to assess the association between centiloid value and [^18^F]‐FEOBV uptake with and without age as a covariate. Group x centiloid interaction was also assessed following the ROI‐based approach. Student's t‐tests, Kendall's Tau rank correlation, GLM, and interaction analyses were performed using R (version 4.4.1). For all ROI‐based analyses, *p* < 0.05, uncorrected and false discovery rate (FDR) corrected for multiple comparisons are shown.

## 3 RESULTS

### 3.1 Adults with display regional increases DS [^18^F]‐FEOBV uptake compared to amyloid‐matched controls

ROI‐based analysis in subject space was performed to compare [^18^F]‐FEOBV uptake between adults with DS and the amyloid‐matched control group. Increased [^18^F]‐FEOBV uptake was observed in adults with DS in the cerebellum, amygdala, thalamus, frontal cortex (superior frontal cortex, caudal middle frontal cortex), temporal cortex (temporal pole, superior temporal cortex), insula cortex, and cingulate cortex (caudal anterior cingulate, rostral anterior cingulate), all *p* < 0.05, FDR‐corrected (Table 2). Kendalls tau rank correlation analysis was performed to compare the rank correlation of [^18^F]‐FEOBV uptake as measured by SUVR across the different ROIs, here the rank ordering of [^18^F]‐FEOBV uptake across regions was found to be very similar (Tau = 0.820, *p*‐value = 4.22 × 10^−15^).

**TABLE 2:** Comparison of SUVRs between adults with DS and amyloid‐matched neurotypically developed adults.

ROI	Control SUVR	Control rank	DS SUVR	DS Rank	*t*‐Value	*p*‐value	FDR corrected *p*‐value
Putamen	8.606	1	9.261	1	−1.694	0.1015	0.2523
Transverse temporal cortex	2.776	2	3.029	4	−1.717	0.0970	0.2523
Amygdala	2.694	3	3.102	3	−3.943	**0.0005**	**0.0023**
Thalamus	2.669	4	3.233	2	−4.55	**<0.0001**	**0.0007**
Caudal anterior cingulate cortex	2.419	5	2.686	5	−2.707	**0.0115**	**0.0426**
Hippocampus	2.333	6	2.382	7	−0.653	0.5192	0.7115
Insula cortex	2.308	7	2.622	6	−5.008	**<0.0001**	**0.0005**
Posterior cingulate cortex	2.235	8	2.197	10	0.457	0.6509	0.7526
Paracentral cortex	2.152	9	2.167	12	−0.143	0.8875	0.9122
Precentral cortex	1.998	10	2.171	11	−2.338	**0.0269**	0.0829
Medial orbitofrontal cortex	1.993	11	2.055	13	−0.881	0.3856	0.5945
Rostral anterior cingulate cortex	1.976	12	2.213	8	−3.912	**0.0005**	**0.0023**
Lateral orbitofrontal cortex	1.931	13	1.92	15	0.171	0.8653	0.9122
Superior temporal cortex	1.893	14	2.206	9	−4.666	**<0.0001**	**0.0007**
Pericalcarine cortex	1.853	15	1.889	17	−0.322	0.7498	0.8160
Pars triangularis	1.758	16	1.815	20	−0.931	0.3597	0.5786
Isthmus of the cingulate gyrus	1.737	17	1.659	27	1.218	0.2334	0.4370
Pars opercularis	1.728	18	1.864	18	−2.365	**0.0252**	0.0829
Entorhinal cortex	1.719	19	1.814	21	−1.211	0.2362	0.4370
Rostral middle frontal cortex	1.696	20	1.779	24	−1.354	0.1865	0.4059
Superior frontal cortex	1.685	21	1.907	16	−4.654	**<0.0001**	**0.0007**
Frontal pole	1.658	22	1.794	23	−1.689	0.1023	0.2523
Supramarginal cortex	1.655	23	1.68	26	−0.466	0.6450	0.7526
Banks of the superior temporal sulcus	1.652	24	1.652	28	−0.011	0.9909	0.9909
Temporal pole	1.643	25	1.801	22	−3.081	**0.0046**	**0.0189**
Caudal middle frontal cortex	1.636	26	1.86	19	−3.899	**0.0005**	**0.0023**
Cerebellum	1.626	27	1.991	14	−5.501	**<0.0001**	**0.0003**
Middle temporal cortex	1.612	28	1.688	25	−1.528	0.1378	0.3187
Inferior temporal cortex	1.598	29	1.631	29	−0.753	0.4577	0.6774
Precuneus	1.555	30	1.574	30	−0.418	0.6794	0.7618
Para hippocampal gyrus	1.522	31	1.567	31	−1.171	0.2515	0.4431
Cuneus	1.494	32	1.421	35	1.078	0.2901	0.4879
Fusiform gyrus	1.479	33	1.499	32	−0.494	0.6255	0.7526
Inferior parietal cortex	1.464	34	1.437	33	0.536	0.5960	0.7526
Superior parietal cortex	1.463	35	1.433	34	−0.088	0.6107	0.7526
Lateral occipital cortex	1.421	36	1.34	37	1.253	0.2206	0.4370
Lingual cortex	1.381	37	1.343	36	0.716	0.4798	0.6828

*Note*: Kendall's Tau rank correlation: Tau = 0.820, *p*−value = 4.22 x 10^−15^.

Abbreviations: DS, Down syndrome; FDR, false discovery rate; ROI, region of interest; SUVR, standard uptake value ration.

Bold values indicate *p* < 0.05 uncorrected and FDR corrected.

### 3.2 [^18^F]‐FEOBV uptake displays regional‐specific reductions associated with increased amyloid burden measured in centiloids

**FIGURE 1 alz70134-fig-0001:**
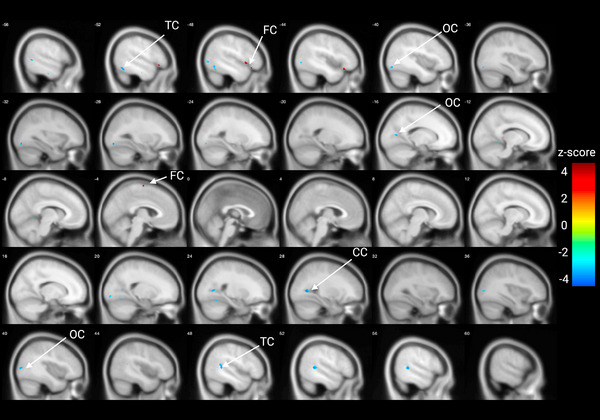
Amyloid is associated with lower [^18^F]‐FEOBV uptake. Increasing amyloid accumulation measured in centiloids is associated with decreasing [^18^F]‐FEOBV uptake in clusters in the cingulate cortex (A), calcarine cortex (B), thalamus (C), right fusiform cortex (D), occipital lobe (E) and right fusiform cortex (F). Cooler colors indicate a negative association between [^18^F]‐FEOBV uptake, no clusters with positive associations were observed. [^18^F]‐FEOBV, [^18^F]‐fluoroethoxybenzovesamicol.

In this cohort of 15 adults with DS, we observe amyloid accumulation in centiloids increasing with age (Figure S1). Numerous cortical clusters display an association between [^18^F]‐FEOBV uptake and global amyloid accumulation when correcting for age (Figure 1; for specific clusters and effect sizes, see Table 3). Specifically, lower [^18^F]‐FEOBV uptake with greater centiloid values was observed in the temporal cortex, occipital cortex, and calcarine cortex, with greater [^18^F]‐FEOBV uptake in frontal cortical regions associated with higher centiloid values. When not correcting for age, lower [^18^F]‐FEOBV uptake in the calcarine cortex, thalamus, temporal lobe, and right fusiform cortex was associated with higher centiloid values (Figure S2 and Table S2). When performing ROI‐based analyses in subject space, a significant association between centiloid and [^18^F]‐FEOBV uptake was observed in the frontal pole (*t* = ‐2.384, *p* = 0.0345), the inferior temporal cortex (*t* = −2.242, *p* = 0.0446), precuneus (*t* = −2.558, *p* = 0.0251) and the lateral occipital cortex (*t* = −2.231, 0.0455) when correcting for age. However, these did not survive corrections for multiple comparisons (Table S3). When not correcting for age, significant associations were observed in the amygdala (*t* = −3.025, *p* = 0.00975), thalamus (*t* = −2.233, 0.0429), posterior cingulate cortex (*t* = −2.244, *p* = 0.040) and the entorhinal cortex (*t* = −2.331, *p* = 0.0365). Again, these did not survive corrections for multiple comparisons (Table S3).

**TABLE 3 alz70134-tbl-0003:** Clusters displaying significant associations between amyloid accumulation in Centiloids and [^18^F]‐FEOBV uptake correcting for age.

Center of MNI coordinate	Cluster region	Cluster size (voxels)	z‐score
−53 –17 –26	Temporal lobe	50	−3.252
−50 –62 –15	Temporal lobe	396	−3.637
−48 25 –13	Frontal lobe	172	3.571
22 –60 –15	Occipital lobe	117	−3.370
−60 –54 –14	Temporal lobe	54	−3.089
−33 –81 –12	Occipital lobe	318	−3.678
21 –90 –6	Occipital lobe	77	−3.253
−48 12 –3	Frontal lobe	72	3.442
−46 –77 –1	Occipital lobe	121	−3.082
39 –82 6	Occipital lobe	187	−3.464
−59 –56 5	Temporal lobe	228	−3.954
26 –62 6	Right calcarine cortex	363	−3.879
51 –44 8	Temporal lobe	675	−3.775
−16 –70 9	Occipital lobe	120	−3.353
−3 –11 71	Frontal lobe	50	3.559

Abbreviations: [^18^F]‐FEOBV, [^18^F]‐fluoroethoxybenzovesamicol; MNI, Montreal Neurological Institute.

### 3.3 Amyloid‐associated effects on [^18^F]‐FEOBV uptake are greater in adults with DS than neurotypically developed individuals

**FIGURE 2 alz70134-fig-0002:**
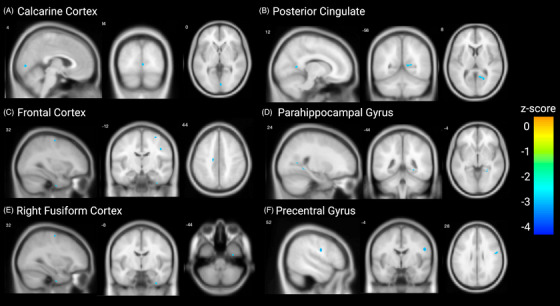
Higher amyloid accumulation is associated with a greater difference in [^18^F]‐FEOBV uptake in adults with DS than neurotypically developed individuals. Clusters in the calcarine cortex (A), posterior cingulate cortex (B), cingulate gyrus (C), parahippocampal gyrus (D), right fusiform cortex (E), and precentral gyrus (F) display a greater amyloid associated difference in [^18^F]‐FEOBV uptake in adults with DS than is observed in neurotypically developed centiloid matched individuals. Cooler colors indicate a greater negative difference in [^18^F]‐FEOBV uptake in adults with DS compared to amyloid‐matched controls. No clusters were found indicating a greater negative difference in [^18^F]‐FEOBV uptake in neurotypically developed adults. DS, Down syndrome; [^18^F]‐FEOBV, [^18^F]‐fluoroethoxybenzovesamicol.

To assess whether adults with DS displayed a greater centiloid‐associated reduction in [^18^F]‐FEOBV uptake than neurotypically developed controls, a centiloid x group interaction (adults with DS or neurotypically developed adults) between the centiloid matched groups was performed. This revealed a number of clusters where a significant interaction was observed (Figure 2, for specific clusters and effect sizes see Table 4; for sagittal sections through the whole brain, see Figure S3). To assess the directionality of these interactions, whole‐brain beta‐values for the centiloid and [^18^F]‐FEOBV uptake association were plotted (Figure S4). Adults with DS displayed clusters with a greater negative difference in [^18^F]‐FEOBV uptake associated with amyloid accumulation in the calcarine cortex, posterior cingulate cortex, cingulate gyrus, parahippocampal gyrus, right fusiform cortex, and the precentral gyrus. By contrast, no clusters revealed a greater negative difference in [^18^F]‐FEOBV uptake in the comparison group of centiloid‐matched neurotypically developed adults.[Fig alz70134-fig-0001], [Table alz70134-tbl-0003]

**TABLE 4 alz70134-tbl-0004:** Clusters displaying significant group by amyloid accumulation interaction effect on [^18^F]‐FEOBV uptake.

Center of MNI coordinate	Cluster region	Cluster size (voxels)	z‐Score
44 –12 36	Frontal lobe	64	−3.050
31 –11 62	Frontal lobe	60	−3.469
34 –9 ‐44	Right fusiform cortex	63	−3.141
25 –44 –5	Parahippocampal gyrus	74	−3.158
3 –83 1	Occipital lobe	98	−3.196
21 –60 9	Posterior cingulate cortex	282	−3.652
54 –4 27	Precentral gyrus	229	−3.374
−14 –17 44	Cingulate gyrus	68	−3.549

Abbreviations: [^18^F]‐FEOBV, [^18^F]‐fluoroethoxybenzovesamicol; MNI, Montreal Neurological Institute.

**FIGURE 3 alz70134-fig-0003:**
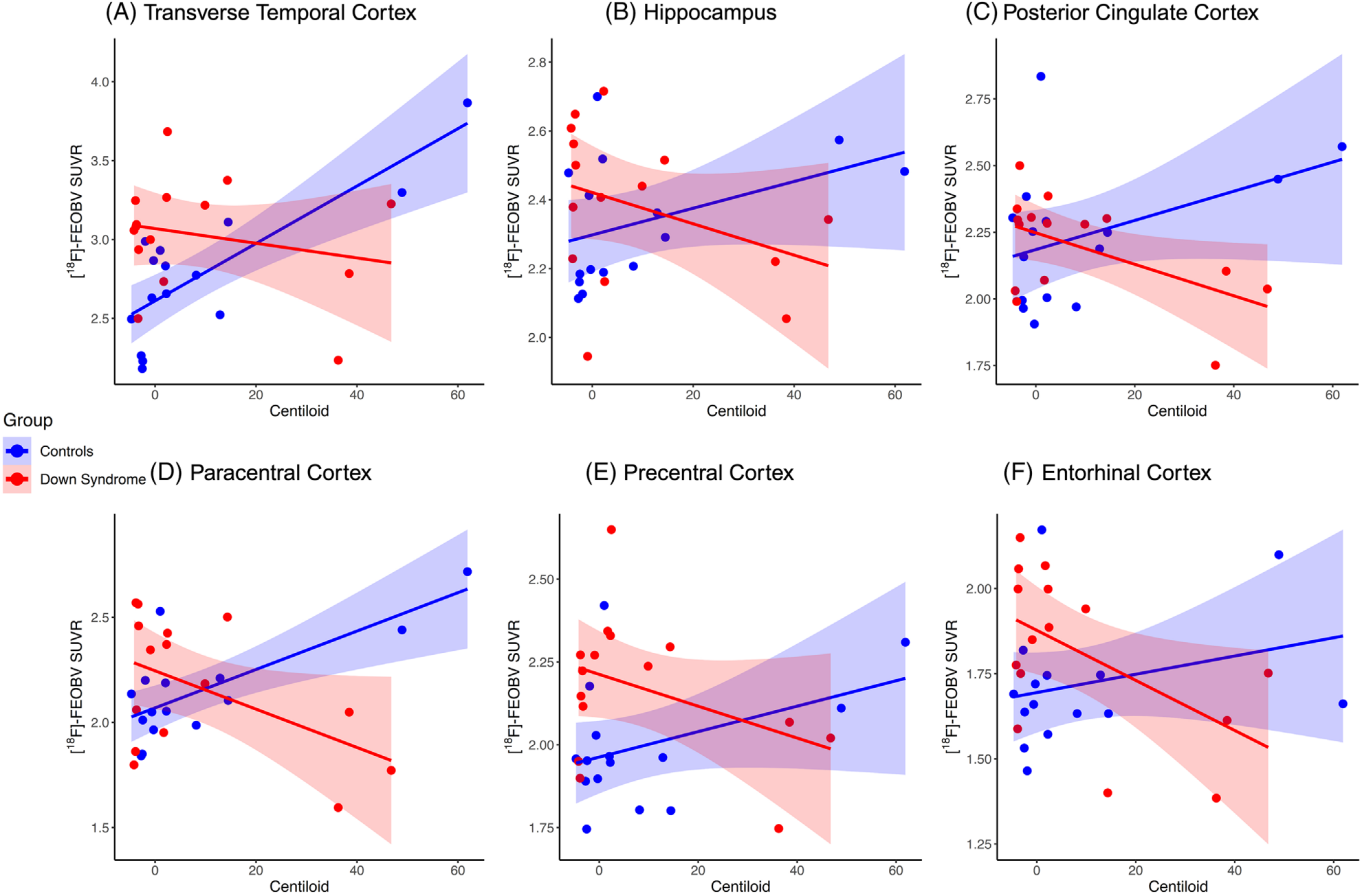
Regions displaying a significant group x amyloid accumulation interaction effect on [^18^F]‐FEOBV uptake. The transverse temporal cortex (A), hippocampus (B), posterior cingulate cortex (C), paracentral cortex (D), precentral cortex (E), and entorhinal cortex (F) displayed a significant group x amyloid accumulation in centiloids interaction on [^18^F]‐FEOBV uptake before correcting for multiple comparisons. The effects observed in the transverse temporal cortex and paracentral cortex survive FDR correction for multiple comparisons. FDR, false discovery rate; [^18^F]‐FEOBV, [^18^F]‐fluoroethoxybenzovesamicol.

Following ROI‐based analyses in subject space, similar regions displayed a significant interaction effect on [^18^F]‐FEOBV uptake between amyloid accumulation in centiloids and group. The transverse temporal cortex (*t* = −3.512, *p* = 0.0017), hippocampus (*t* = −2.101, *p* = 0.0454), posterior cingulate cortex (*t* = −2.676, *p* = 0.0127), paracentral cortex (*t* = −3.760, *p* = 0.0009), precentral cortex (*t* = −2.185, *p* = 0.0381), and entorhinal cortex (*t* = −2.476, *p* = 0.0201) displayed a significant interaction effect, with the effects observed in the transverse temporal cortex and paracentral cortex surviving FDR correction for multiple comparisons (Figure 3 and Table S3).

### 3.4 Cognitive performance in adults with DS associated with region‐specific [^18^F]‐FEOBV uptake

[^18^F]‐FEOBV uptake was associated with performance across three cognitive tasks: mCRT (higher score is better), DSMSE (higher score is better), and Stroop Cats and Dogs switch and accuracy composite score (lower value is better).

**FIGURE 4 alz70134-fig-0004:**
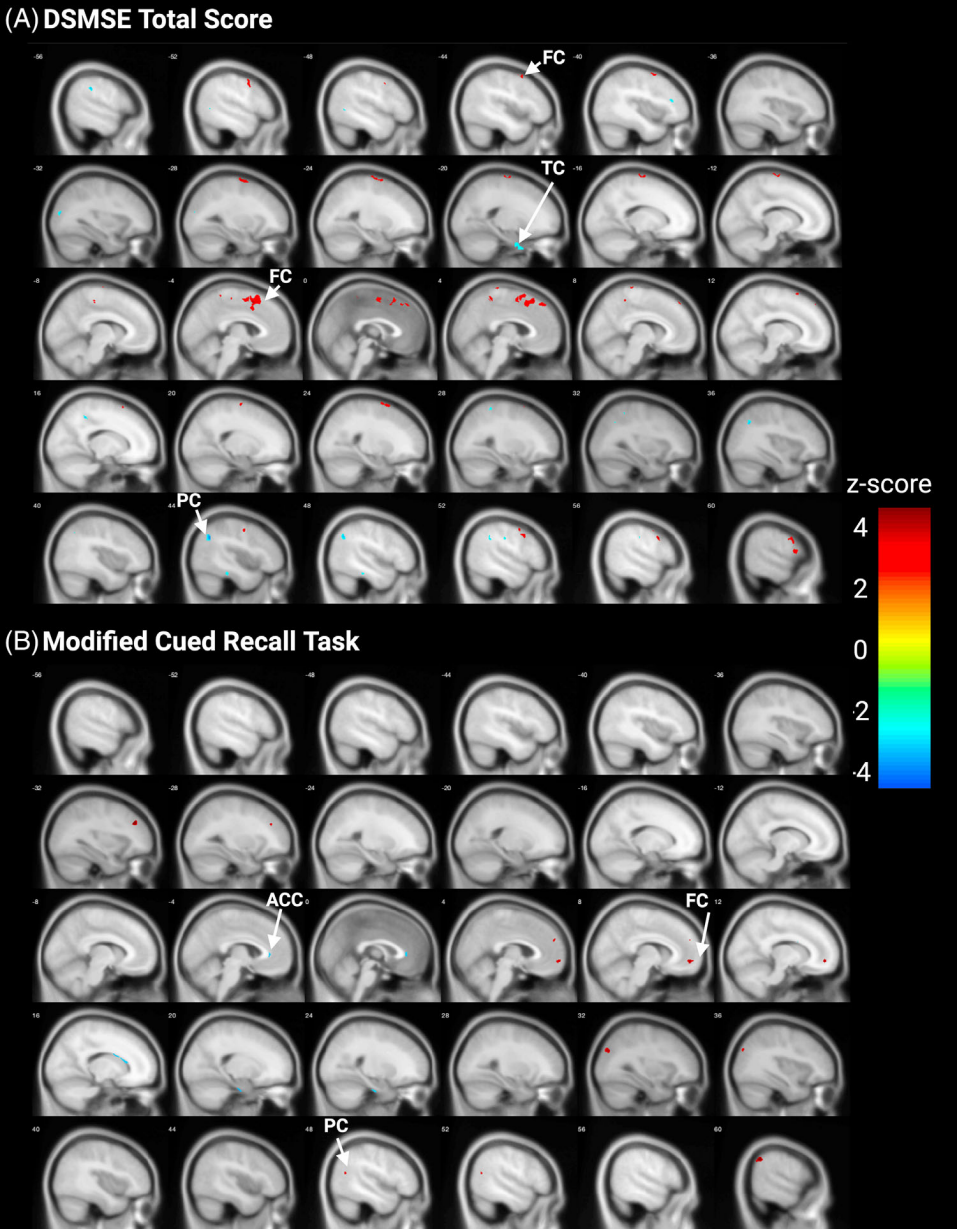
Associations between [^18^F]‐FEOBV uptake and cognitive performance. [^18^F]‐FEOBV uptake displays predominantly positive associations with performance on the DSMSE task, with discrete posterior and temporal clusters displaying negative associations (A). [^18^F]‐FEOBV uptake displays positive and negative associations in cortical clusters on the mCRT (B). Hotter colors indicate a positive association between [^18^F]‐FEOBV uptake and cognitive score, cooler colors indicate a negative association. For both tasks, a higher score indicates better performance. AC, anterior cingulate cortex; DSMSE, Down Syndrome Mental State Examination; FC, frontal cortex; [^18^F]‐FEOBV, [^18^F]‐fluoroethoxybenzovesamicol; mCRT, modified Cued Recall Task; PC, parietal cortex; TC, temporal cortex.

The total score on the DSMSE was positively associated with [^18^F]‐FEOBV uptake in multiple larger clusters in the frontal lobe and one cluster in each of the parietal lobe and insular cortex. The total score on DSMSE was negatively associated with [^18^F]‐FEOBV uptake in clusters in the temporal, occipital, frontal, and parietal lobes (Figure 4A). [^18^F]‐FEOBV uptake was positively associated with total free recall in the mCRT in clusters in the frotal and parietal lobes and negatively associated wth clusters in the occipital lobe, limbic lobe, frontal lobe, parietal lobe and brain stem (Figure 4B) Similar clusters are observed when utilizing a DS‐specific template (Figure S5). When separating the DSMSE into memory and non‐memory sub‐scores, the non‐memory sub‐scores positively associated with [^18^F]‐FEOBV uptake in clusters in the frontal lobe and negatively associated with [^18^F]‐FEOBV uptake in clusters in the temporal, occipital, frontal, parietal, and limbic lobes (Figure S6A). The memory sub‐score displayed only positive associations with [^18^F]‐FEOBV uptake, with clusters observed in the temporal lobe, occipital lobe, insular, frontal lobe, and parietal lobe (Figure S6B). Similar significant clusters were observed following analyses on a DS‐specific template (Figure S7).

Stroop Cats and Dogs switch time and accuracy composite score negatively associated with [^18^F]‐FEOBV uptake in clusters in the temporal, frontal, and limbic lobes and fusiform gyrus, and positively associated with [^18^F]‐FEOBV uptake in clusters in the parietal and occipital lobes in both MNI‐152 and DS‐specific template space (Figure S8).

## 4 DISCUSSION

Previous studies in sporadic AD have demonstrated that [^18^F]‐FEOBV uptake is lower with disease progression and is associated with amyloid accumulation.^24^ Global cortical [^18^F]‐FEOBV uptake was found to be lower in cognitively unimpaired individuals with higher amyloid accumulation.^9^ Studies of the cholinergic system in adults with DS during AD development have focused on the cholinergic basal forebrain at postmortem,^2^, ^51^ or using MRI volumetry, demonstrating declining cholinergic basal forebrain volume with increasing AD pathology.^17^ The present study is the first to assess the cholinergic system in adults with DS in relation to AD pathology using [^18^F]‐FEOBV PET.[Fig alz70134-fig-0002], [Table alz70134-tbl-0004], [Fig alz70134-fig-0003], [Fig alz70134-fig-0004]

Similar to our previous work,^25^ we observe that adults with DS display increased [^18^F]‐FEOBV uptake compared to controls. In this study, the control group was amyloid‐matched, in contrast to our previous study, in which the control group was age‐matched. To enable amyloid‐matching, the controls group is older than the adults with DS. With negative relationships between age and [^18^F]‐FEOBV uptake reported,^19^, ^20^ these changes could be age‐related. However, the effects observed are similar to our comparison of adults with DS to age‐matched controls. The [^18^F]‐FEOBV uptake SUVR rank order across different regions was assessed and found to be highly correlated between adults with DS and the amyloid‐matched control group, suggesting that, although some areas display higher [^18^F]‐FEOBV uptake, the general uptake pattern is similar between groups.

Exploratory analysis suggests that age‐corrected [^18^F]‐FEOBV uptake in posterior cortical clusters was lower in individuals with higher global amyloid, while [^18^F]‐FEOBV uptake in frontal clusters was higher in individuals with higher global amyloid. This may suggest compensatory responses in frontal areas in non‐demented adults with DS, with decline in more posterior regions; however, further longitudinal studies will be required to test this hypothesis. When assessing the group x amyloid interaction, numerous clusters displayed a greater reduction in [^18^F]‐FEOBV uptake associated with amyloid accumulation in adults with DS than amyloid‐matched neurotypically developed adults. ROI‐based analyses displayed significant interactions in similar regions. In all regions that reached significance, a positive association between global amyloid and [^18^F]‐FEOBV uptake in amyloid‐matched controls and a negative association in non‐demented adults with DS was observed. These cross‐sectional findings suggest, in preclinical AD, neurotypically developed individuals compensate for developing AD pathology with increased VAChT expression, whereas adults with DS show a decline in cholinergic terminal density with early AD pathology. There are several reasons for this potential more rapid decline in [^18^F]‐FEOBV uptake in adults with DS. Adults with DS display a shorter interval between amyloid and tau accumulation than neurotypically developed individuals^52^, ^53^; this may suggest that this shorter timeframe leads to a reduction in cholinergic terminals. Supporting this, previous studies indicate tau accumulation is associated with cholinergic decline.^15^, ^16^ Alternatively, previous studies have suggested that an association between amyloid accumulation and cholinergic degeneration^12^, ^13^ could suggest an increased susceptibility to amyloid‐related cholinergic loss in adults with DS compared to the neurotypically developed population. The trophic support for cholinergic neurons is provided by nerve growth factor (NGF), which displays metabolic dysregulation following amyloid accumulation.^54^ Recent studies indicate that non‐demented adults with DS display similar NGF metabolism dysregulation.^55^ This disrupted NGF metabolism may underlie increased susceptibility to amyloid‐related cholinergic pathology.

To investigate whether these cholinergic changes were linked to changes in cognition, we investigated the relationship between cognitive performance with [^18^F]‐FEOBV uptake. The mCRT is a task testing recall memory and better recall was associated with higher and lower [^18^F]‐FEOBV uptake across different cortical clusters. Previous studies have shown a positive association between cholinergic basal forebrain volume and mCRT performance in a cohort of adults with DS, including individuals who are non‐demented and demented.^17^ However, studies with radiotracers targeting the VAChT assessing cognition in different syndromes have been less conclusive. In schizophrenia, regional vesicular acetylcholine transporter‐specific tracer (‐)‐(1‐(8‐(2‐[^18^F]fluoroethoxy)‐3‐hydroxy‐1,2,3,4‐tetrahydronaphthalen‐2‐yl)‐piperidin‐4‐yl)(4‐fluorophenyl)methanone ([^18^F]‐VAT) uptake was found to negatively associate with an attention task,^56^ while in mild cognitive impairment (MCI) cortical [^18^F]‐FEOBV uptake was found to positively associate with tasks of attention, executive function, and language.^9^ These data highlight a potentially complex relationship between VAChT binding and cognitive performance in different disease states. The DSMSE is a battery that assesses global cognition. The total score was predominantly positively associated with [^18^F]‐FEOBV uptake, an effect seen in both the memory and non‐memory sub‐scores. The non‐memory sub‐score is also negatively associated with [^18^F]‐FEOBV uptake in posterior clusters. This is similar to previously published work, where global cognition on the CAMCOG‐DS (Cambridge Examination for Mental Disorders of Older People with DS) was positively associated with basal forebrain volume.^17^ These positive associations between [^18^F]‐FEOBV uptake and global cognition in non‐demented adults with DS may suggest that individuals with DS display reduced cognitive reserve, and thus, a small insult to the cholinergic system can result in detectable changes in cognition. Finally, the Stroop Cats and Dogs switch time showed clusters in frontal and limbic regions where a lower composite score (i.e., faster [shorter] switch times and/or fewer errors) was associated with higher [^18^F]‐FEOBV uptake, while small clusters across parietal and occipital regions show a higher composite score (i.e., slower [longer] switch times and/or increased errors) associated with higher [^18^F]‐FEOBV uptake. The data across these three tasks suggests that during the development of early AD pathology, the DSMSE global cognitive assessment may be the best correlate of cortical cholinergic terminal density in individuals with DS. Further longitudinal assessments will be necessary to fully elucidate the relationship between [^18^F]‐FEOBV uptake and cognitive performance in adults with DS.

This study has several limitations that are important to consider. When comparing the adults with DS to the control group, there are age and sex distribution differences between the two groups, which may underlie some of the differences in [^18^F]‐FEOBV uptake; however, these results are similar to the difference in [^18^F]‐FEOBV uptake we observed between adults with DS and age‐matched neurotypically developed individuals. Second, this study focuses on amyloid pathology with no parallel assessment of tau pathology. This limitation is due to data availability; only eight of the participants with DS had tau imaging data available, and there were few participants with significant tau accumulation. Third, the amyloid‐matched neurotypically developed comparison group is an all‐female cohort, and both cohorts are predominantly white. Regarding the all‐female amyloid‐matched control cohort, previous studies in sporadic AD have indicated no sex differences in the association between amyloid pathology and cholinergic basal forebrain volume.^12^, ^57^ To address the racial homogeneity in the DS cohort, efforts are being made to recruit participants from under‐represented populations as this cohort develops. Fourth, as this cohort is non‐demented adults with DS, there were only three participants that had a centiloid value greater than 20, with two additional participants with centiloid values of 9.9 and 14.3; the remaining participants had a centiloid value < 4. This low number of participants with high brain amyloid and large number of comparisons may lead to increased type 1 (false positive) statistical error in the exploratory analyses. To limit type 1 statistical error, the *p*‐value was adjusted to *p* < 0.005 for the voxel‐based analyses, while FDR and non‐FDR corrected *p*‐values were reported for ROI‐based analyses. For voxel‐based analyses, the [^18^F]‐FEOBV SUVR was thresholded to > 1 to reduce the number of comparisons and remove areas of nonspecific binding. Fifth, the amyloid‐matched control group is markedly older than the adults with DS, due to AD pathology developing later in the neurotypically developed population. As such, it is not possible to correct for age when performing comparisons between the cohorts. Amyloid accumulation occurs in a manner tightly linked to increasing age in adults with DS, however, as there are previous age‐related changes in [^18^F]‐FEOBV uptake, it is important to consider the effect of age as a covariate, thus we have reported the association between [^18^F]‐FEOBV uptake and amyloid accumulation with and without age as a covariate. Finally, associations between [^18^F]‐FEOBV uptake and cognitive performance were not assessed in the amyloid‐matched control group. Previous studies in cognitively normal controls have indicated no associations between cognitive performance and a similar VAChT radiotracer.^56^ Additionally, the cognitive assessments performed in the amyloid‐matched control group are very different tasks, making group‐wise comparisons difficult.

In conclusion, we have demonstrated that [^18^F]‐FEOBV uptake is increased in adults with DS compared to neurotypical controls; however, the pattern of [^18^F]‐FEOBV uptake across the brain is similar between groups. Exploratory analyses suggest higher amyloid accumulation is associated with predominantly lower [^18^F]‐FEOBV uptake in adults with DS, with AD pathology potentially producing cholinergic compensation in non‐demented neurotypically developed individuals. Further longitudinal studies will be necessary to validate these findings. [^18^F]‐FEOBV uptake is associated with performance on cognitive tasks in individuals with DS, particularly in the DSMSE. Studies such as this will help clarify the relationship between developing Alzheimer's disease, changing cholinergic function, and cognitive performance and may contribute to the development and assessment of novel therapeutic strategies in individuals with DS. Future studies will longitudinally follow the relationship between AD‐related pathology and measures of cholinergic system integrity in individuals with DS to understand the disease dynamics and their impact on cholinergic structure and function.

## CONFLICT OF INTEREST STATEMENT

Dr. Newhouse and Dr. Dumas report grants from National Institute on Aging, during the conduct of the study; Dr. Rafii reports personal fees from AC Immune, Alzheon, Aptah Bio, Biohaven, Keystone Bio, Positrigo, Ionis, grants from Eisai and Lilly, outside the submitted work; Dr. Rosenberg reports grants and nonfinancial support from General Electric Healthcare, outside the submitted work; the remaining authors have no conflicts to disclose. Author disclosures are available in the Supporting Information.

## CONSENT STATEMENT

All participants or their legally authorized representatives for the respective studies and the associated cholinergic PET substudies gave written informed consent in accordance with the Declaration of Helsinki and approved institutional review board protocol.

## Supporting information



Supporting Information

Supporting Information
