# Supplementary material for: Brain cholinergic terminal density utilizing [18F]‐fluoroethoxybenzovesamicol PET in adults with Down's syndrome: Relationship to amyloid PET and cognitive performance
Source: Alzheimers Dement. 2025 Apr 6;21(4):e70134. doi: 10.1002/alz.70134 (PMC11972980; doi:10.1002/alz.70134)
Supplement: Supplementary file 1 — Supporting Information [file ALZ-21-e70134-s001.docx]

**Supplemental Figures and Tables**


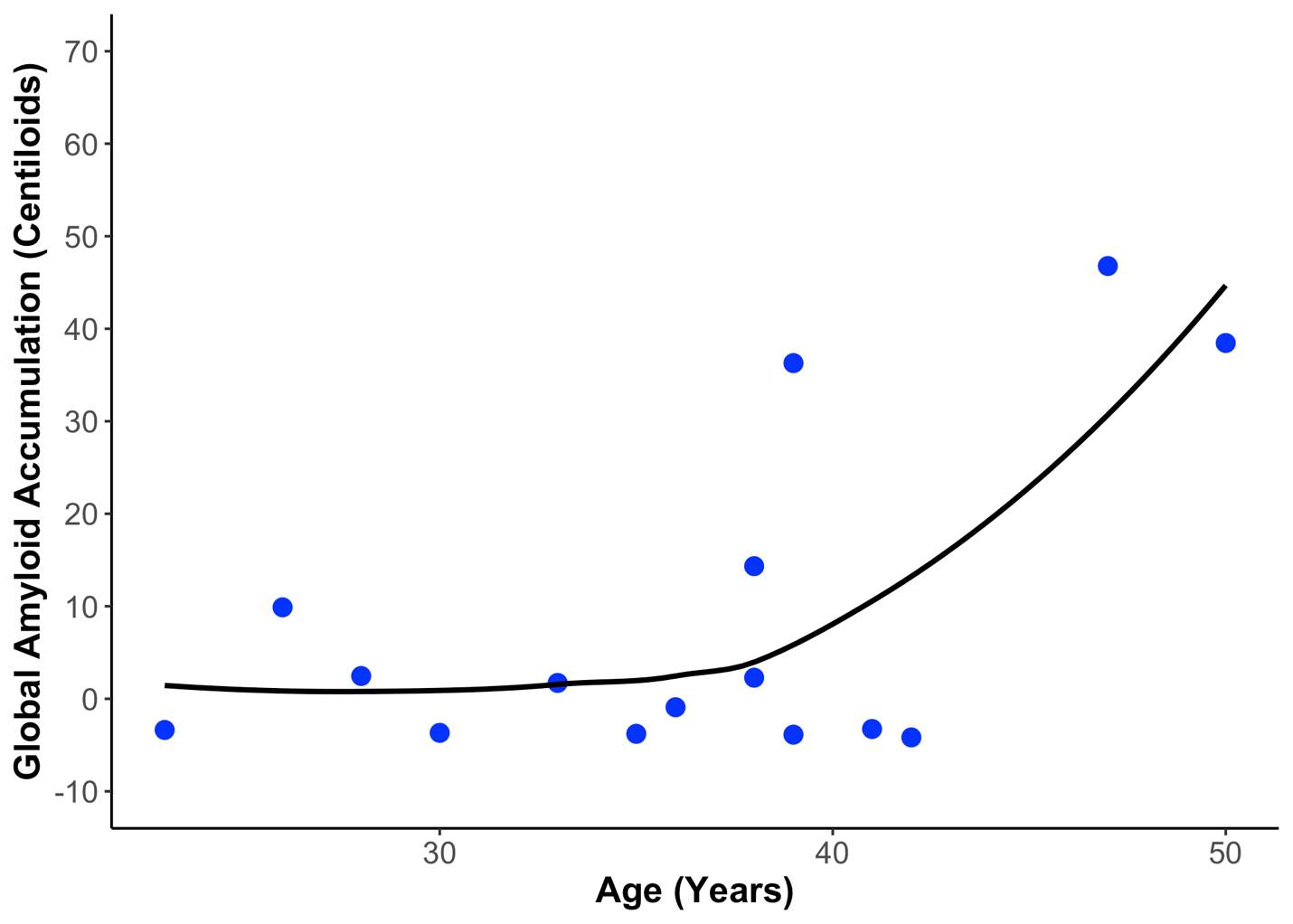
 **Supplementary Figure 1. Amyloid accumulation with increasing age.** Amyloid measured in centiloids (y-axis) increases with age (x-axis) in adults with Down syndrome. The curve is a smoothed representation of the relationship between age and amyloid accumulation generated using Locally Estimated Scatterplot Smoothing (LOESS) with a span of 1.


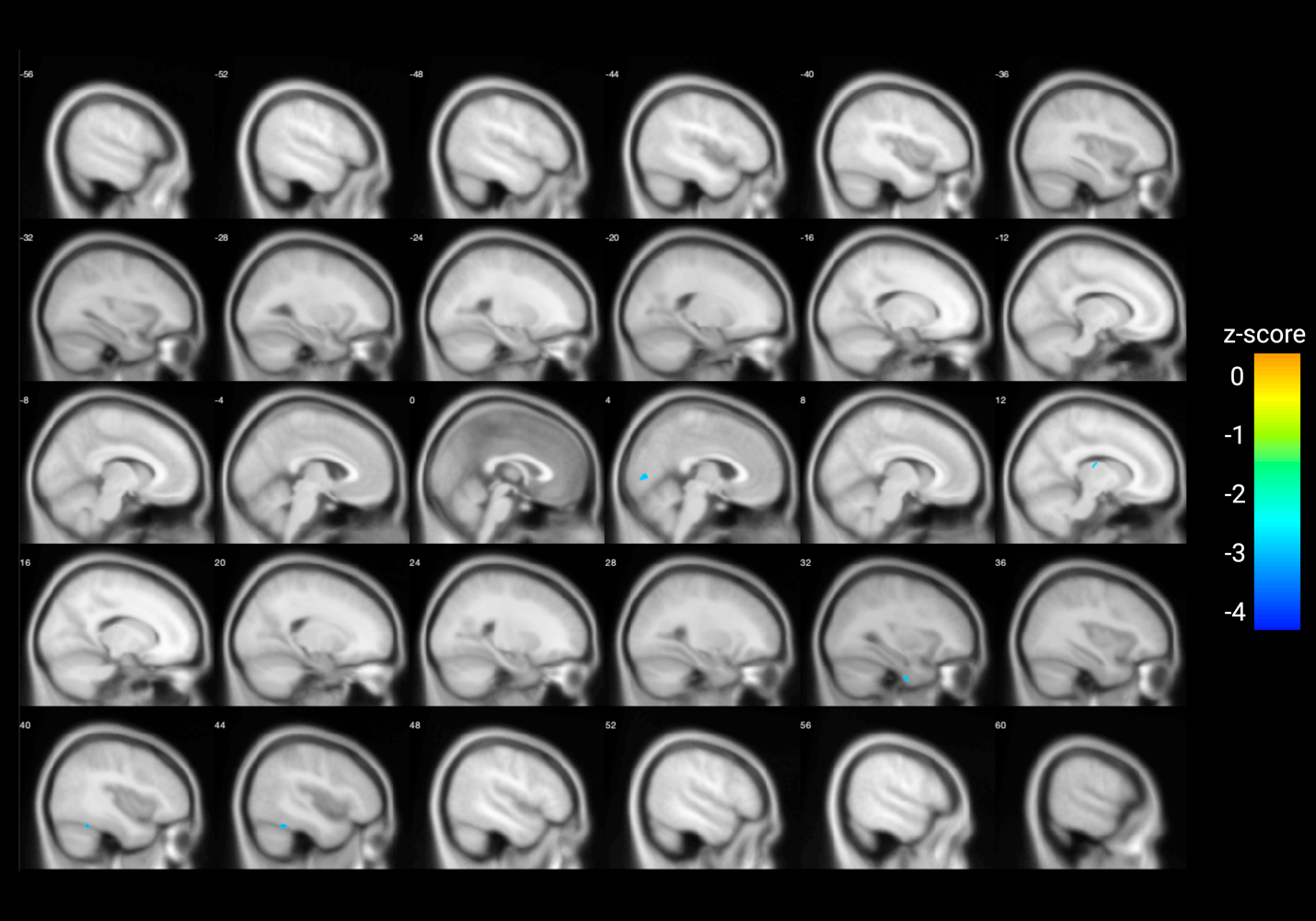


**Supplementary Figure 2. Amyloid associations with [^18^F]-FEOBV uptake through the whole brain.** Shown are sagittal sections through the whole brain showing clusters where [^18^F]-FEOBV uptake associates with amyloid accumulation in centiloids. Cooler colors indicate a negative association between [^18^F]-FEOBV uptake, no clusters with positive associations were observed.


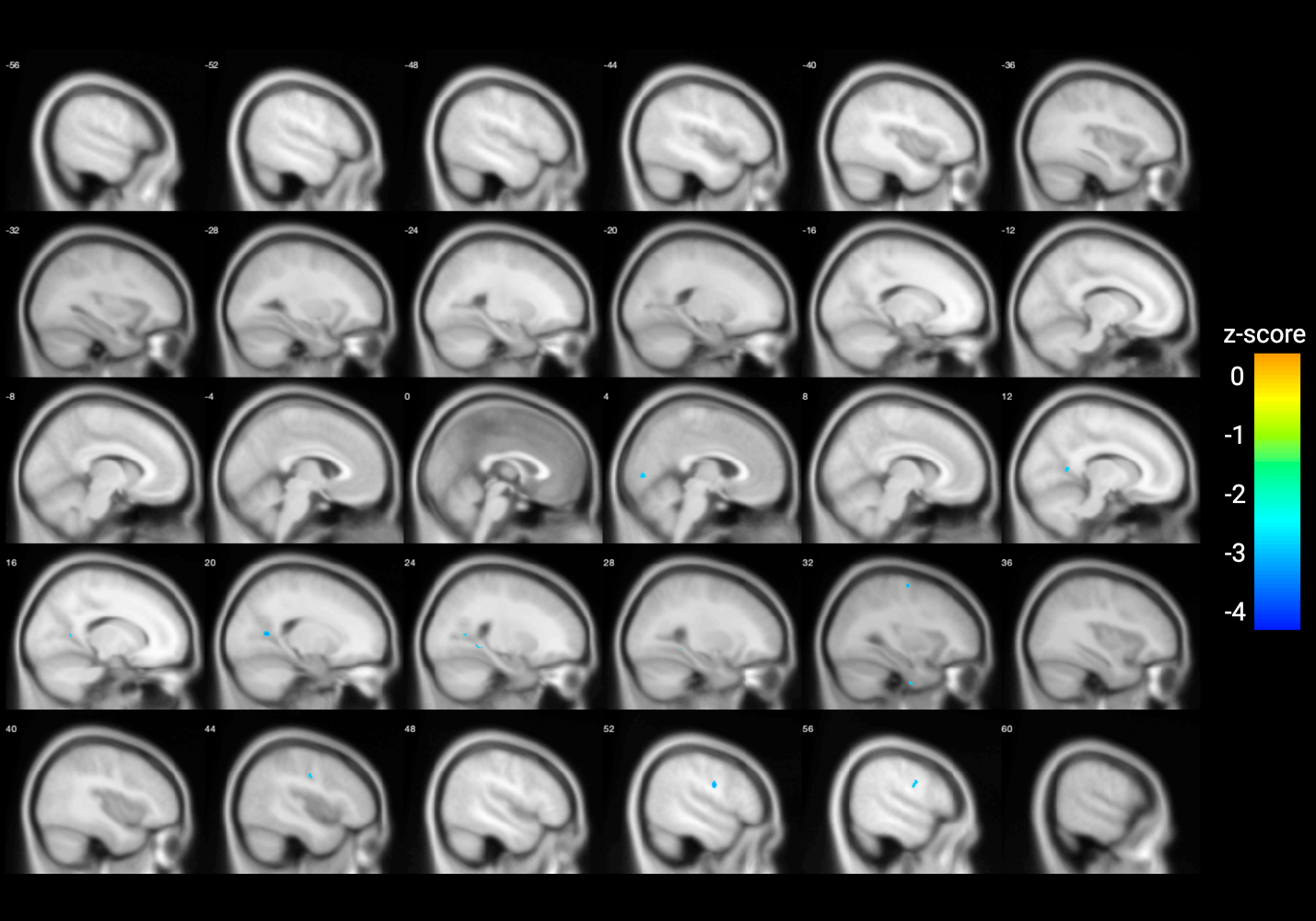


**Supplementary Figure 3. Amyloid x group interaction with [^18^F]-FEOBV uptake through the whole brain.** Shown are sagittal sections through the whole brain showing clusters where there is a significant amyloid x group interaction on [^18^F]-FEOBV uptake. Cooler colors indicate a greater amyloid-associated decrease in adults with DS. No clusters were found indicating a greater amyloid-associated decrease in neurotypically developed adults.


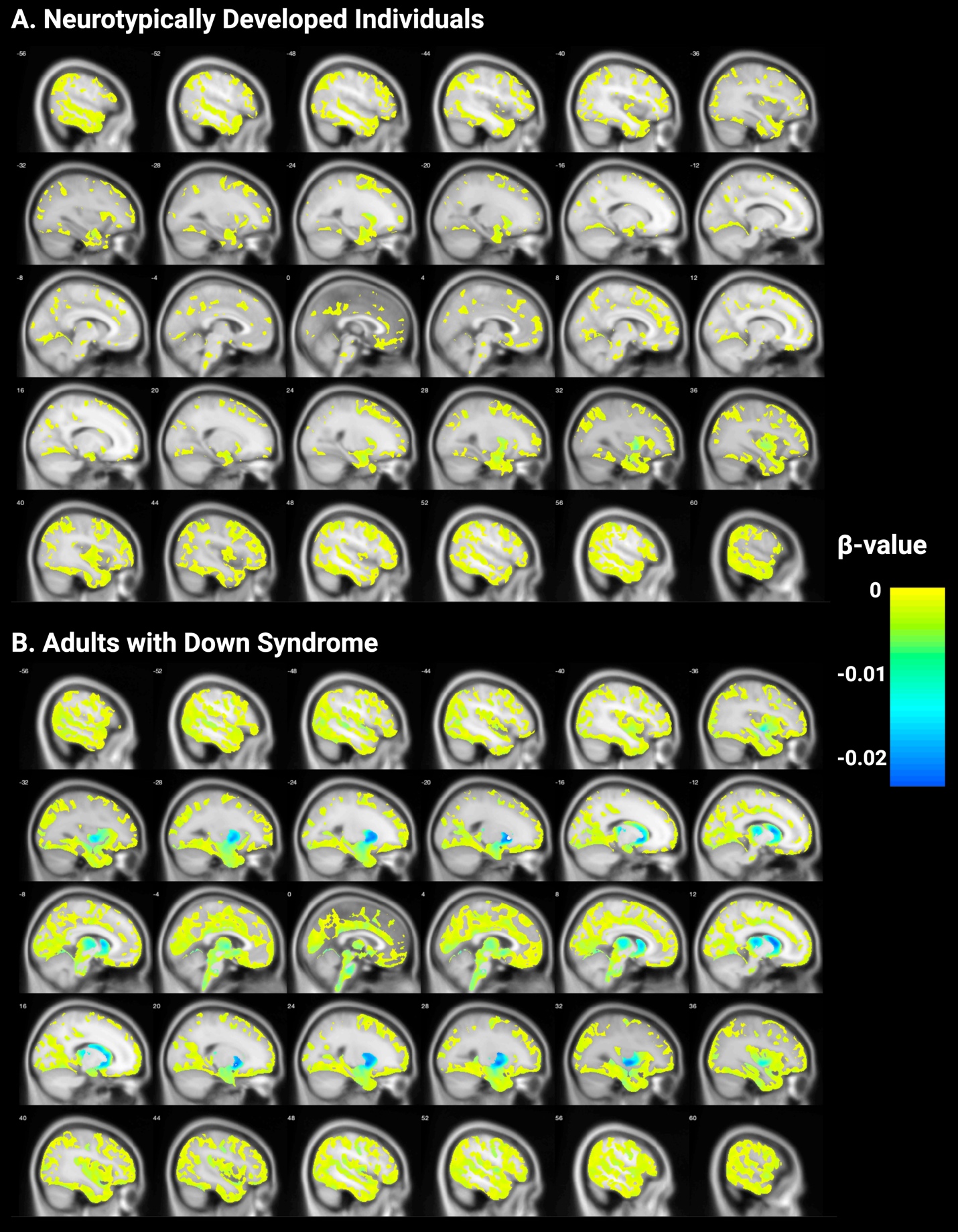


**Supplementary Figure 4. Amyloid-associated differences in [^18^F]-FEOBV uptake.** Shown are the negative beta-values of the whole brain (minus white matter and cerebellum) voxel-based association between amyloid accumulation and [^18^F]-FEOBV uptake in neurotypically developed individuals (A) and adults with DS (B). Yellow indicates a beta value of zero, with colder colors indicating lower [^18^F]-FEOBV uptake with higher amyloid accumulation. All negative voxels are shown with no masking by significance performed.


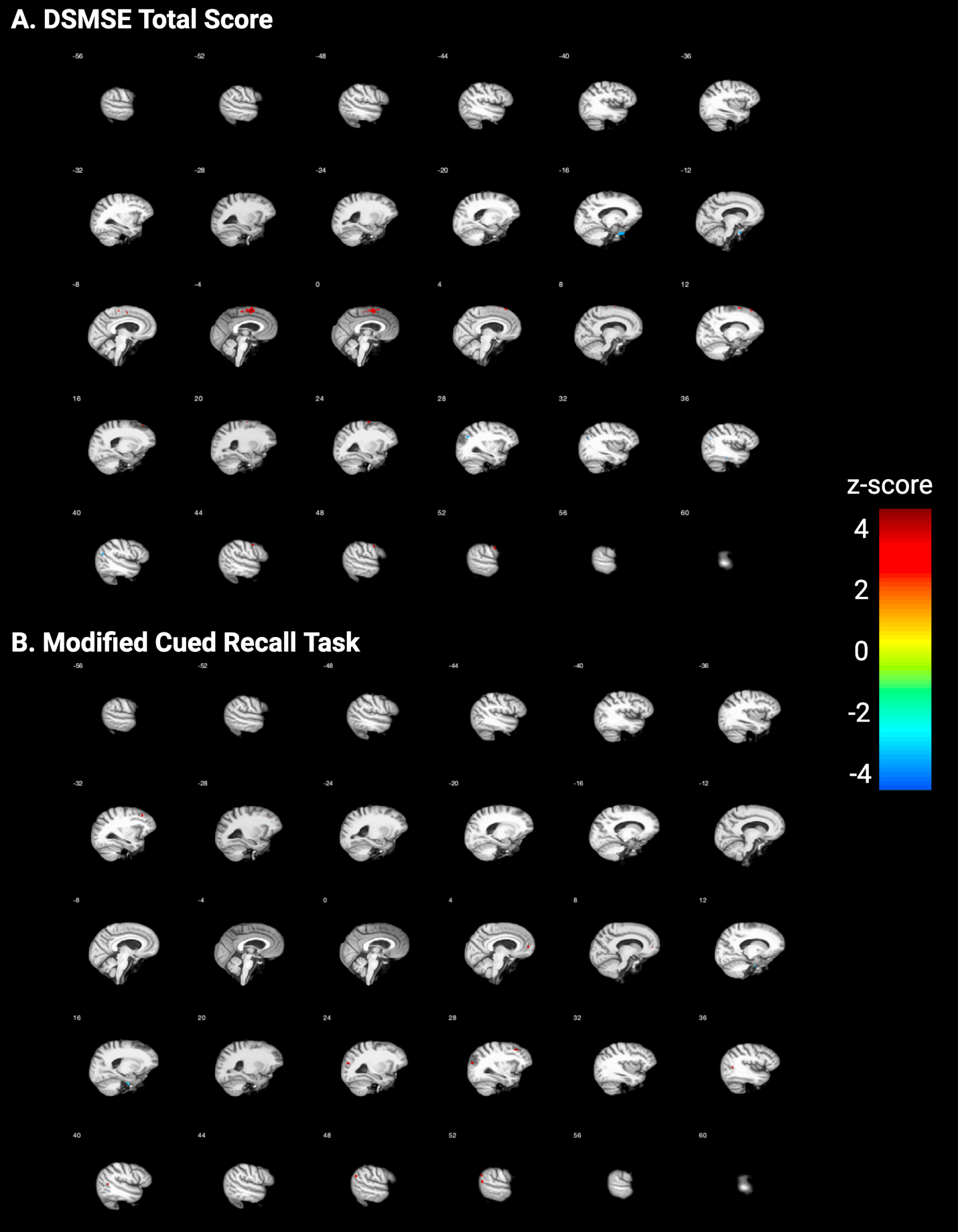


**Supplementary Figure 5. Associations between [^18^F]-FEOBV uptake and cognitive performance on the DSMSE and mCRT following transposing to a Down syndrome specific template.** [^18^F]-FEOBV uptake displays positive and negative associations in cortical and subcortical clusters on the modified cued recall task (A). [^18^F]-FEOBV uptake displays predominantly positive associations with performance on the DSMSE task, with discrete posterior clusters displaying negative associations (B). Hotter colors indicate a positive association between [^18^F]-FEOBV uptake and cognitive score, cooler colors indicate a negative association. For both tasks, a higher score indicates better performance. AC, Anterior Cingulate Cortex; BS, Brain Stem; FC, Frontal Cortex; IC, insular cortex; PC, parietal cortex; PHG, parahippocampal gyrus; TC, temporal cortex

**
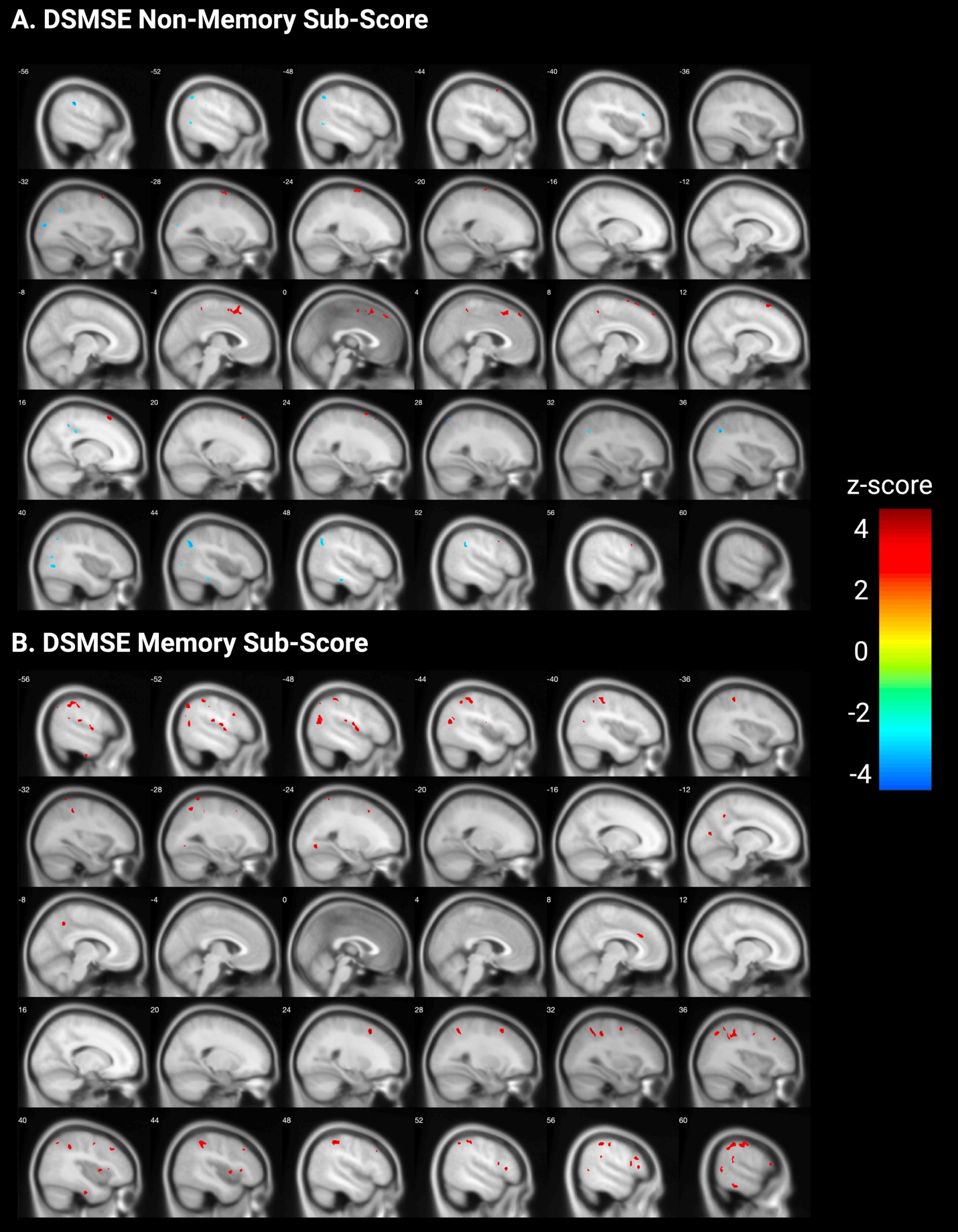
**

**Supplementary Figure 6. Associations between [^18^F]-FEOBV uptake and DSMSE sub-scores** [^18^F]-FEOBV uptake display positive and negative associations in cortical clusters with the non-memory sub-score of the DSMSE (A). [^18^F]-FEOBV uptake displays positive associations with performance on the memory sub-score of the DSMSE task (B). Hotter colors indicate a positive association between [^18^F]-FEOBV uptake and cognitive score, cooler colors indicate a negative association. A higher score indicates better performance.


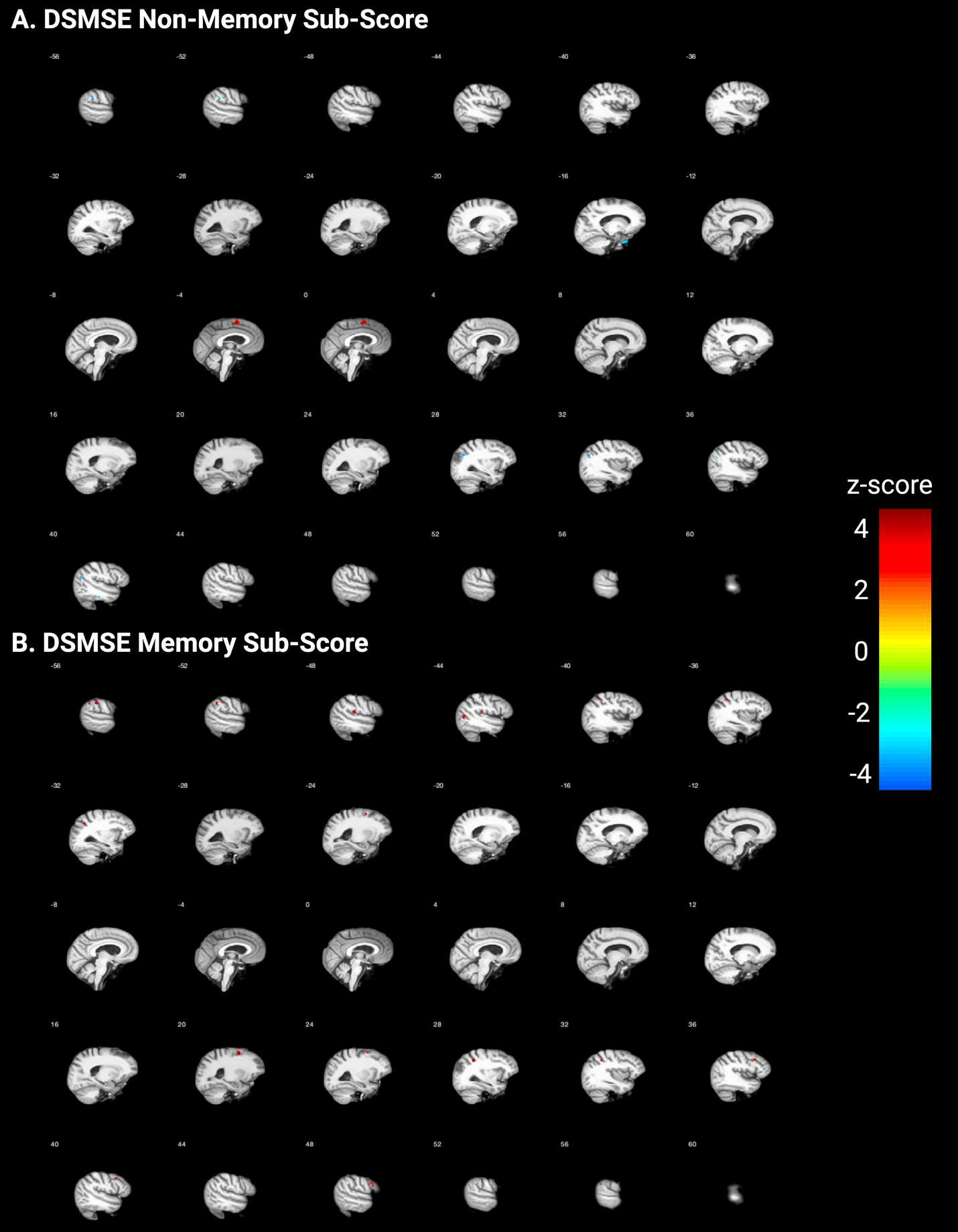


**Supplementary Figure 7. Associations between [^18^F]-FEOBV uptake and DSMSE sub-scores following transposing to a Down syndrome-specific template.** [^18^F]-FEOBV uptake display positive and negative associations in cortical clusters with the non-memory sub-score of the DSMSE when analysed in DS-template space (A). [^18^F]-FEOBV uptake displays positive associations with performance on the memory sub-score of the DSMSE task when analysed in DS-template space (B). Hotter colors indicate a positive association between [^18^F]-FEOBV uptake and cognitive score, cooler colors indicate a negative association. A higher score indicates better performance.


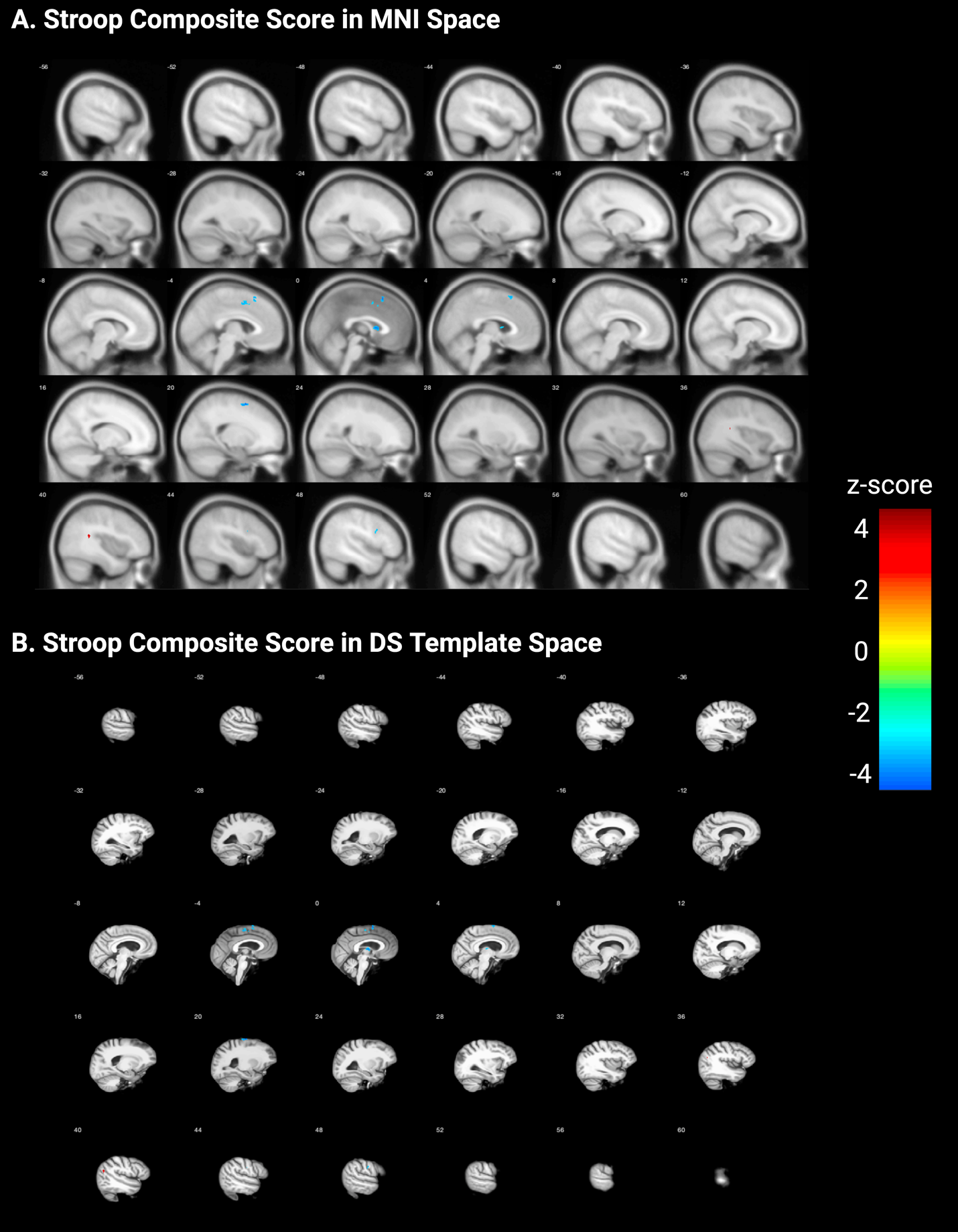


**Supplementary Figure 8. Associations between [^18^F]-FEOBV uptake and Cats and Dogs Stroop switch time** [^18^F]-FEOBV uptake displays positive and negative associations in cortical clusters with the Cats and Dogs Stroop switch time in MNI space (A) and following transposing to a Down syndrome specific template (B). Hotter colors indicate a positive association between [^18^F]-FEOBV uptake and cognitive score, cooler colors indicate a negative association. A lower score indicates better performance, i.e., a quicker switch time.

**Supplementary Table 1. Centiloid Matching**

|  | **Adults with Down Syndrome** | | **Neurotypically Developed Adults** | |
| --- | --- | --- | --- | --- |
| Participant | Age | Centiloid Value | Age | Centiloid Value |
| 1 | 42 | -2.87 | 56 | -2.74 |
| 2 | 38 | 14.34 | 56 | 14.48 |
| 3 | 47 | 63.57 | 69 | 61.89 |
| 4 | 36 | -0.92 | 63 | -0.30 |
| 5 | 50 | 38.46 | 68 | 48.93 |
| 6 | 30 | -3.68 | 65 | -2.44 |
| 7 | 38 | 2.28 | 56 | 2.06 |
| 8 | 26 | 9.89 | 61 | 8.16 |
| 9 | 35 | -3.79 | 63 | -2.53 |
| 10 | 39 | 36.29 | 60 | 12.87 |
| 11 | 39 | -3.87 | 58 | -0.63 |
| 12 | 41 | -3.26 | 59 | -4.68 |
| 13 | 28 | 2.46 | 53 | 1.02 |
| 14 | 33 | 1.72 | 69 | 2.24 |
| 15 | 23 | -3.37 | 67 | -1.93 |

**Supplementary Table 2. Clusters Displaying Significant Associations Between Amyloid Accumulation in Centiloids and [^18^F]-FEOBV uptake.**

| **Center of MNI  co-ordinate** | **Cluster region** | **Cluster size (voxels)** | **z-score** |
| --- | --- | --- | --- |
| 33 -9 -41 | Right Fusiform Cortex | 125 | -3.270 |
| 43 -51 -22 | Right Fusiform Cortex | 200 | -3.505 |
| 4 -83 2 | Calcarine Cortex | 129 | -3.177 |
| 64 -9 -31 | Temporal Lobe | 65 | -3.378 |
| 14 -17 15 | Thalamus | 55 | -3.158 |

**Supplementary Table 3. ROI-based Associations Between [^18^F]-FEOBV Uptake and Centiloid**

|  | **Centiloid (age controlled)** | | | **Centiloid** | | | **Centiloid x Group Interaction** | | |
| --- | --- | --- | --- | --- | --- | --- | --- | --- | --- |
| **ROI** | **t-value** | **p-value** | **FDR-corrected** | **t-value** | **p-value** | **FDR-corrected** | **t-value** | **p-value** | **FDR-corrected** |
| Putamen | -1.218 | 0.246 | 0.423 | -1.486 | 0.161 | 0.439 | -1.352 | 0.188 | 0.308 |
| Transverse Temporal Cortex | -0.859 | 0.407 | 0.505 | -0.838 | 0.417 | 0.577 | -3.512 | **0.0017** | **0.030** |
| Amygdala | -1.857 | 0.0881 | 0.317 | -3.025 | **0.00975** | 0.351 | -1.704 | 0.100 | 0.264 |
| Thalamus | -1.644 | 0.126 | 0.324 | -2.244 | **0.0429** | 0.386 | -1.871 | 0.0727 | 0.264 |
| Caudal Anterior Cingulate Cortex | -0.929 | 0.317 | 0.456 | -0.671 | 0.514 | 0.685 | -1.858 | 0.0746 | 0.264 |
| Hippocampus | -0.890 | 0.391 | 0.503 | -1.372 | 0.193 | 0.439 | -2.101 | **0.0454** | 0.264 |
| Insula Cortex | -1.044 | 0.317 | 0.456 | -1.604 | 0.133 | 0.439 | -1.76 | 0.0902 | 0.264 |
| Posterior Cingulate Cortex | -1.566 | 0.143 | 0.343 | -2.282 | **0.040** | 0.386 | -2.676 | **0.0127** | 0.152 |
| Paracentral Cortex | -1.218 | 0.247 | 0.423 | -2.063 | 0.0597 | 0.430 | -3.76 | **0.0009** | **0.030** |
| Precentral Cortex | -0.436 | 0.670 | 0.731 | -1.504 | 0.156 | 0.439 | -2.185 | **0.0381** | 0.264 |
| Medial Orbitofrontal Cortex | -0.820 | 0.438 | 0.526 | 0.937 | 0.357 | 0.559 | -0.968 | 0.342 | 0.440 |
| Rostral Anterior Cingulate Cortex | -1.267 | 0.229 | 0.423 | -0.838 | 0.417 | 0.577 | -1.555 | 0.132 | 0.264 |
| Lateral Orbitofrontal Cortex | -0.944 | 0.363 | 0.484 | -0.092 | 0.928 | 0.965 | -1.048 | 0.304 | 0.405 |
| Superior Temporal Cortex | -1.243 | 0.238 | 0.423 | -1.028 | 0.323 | 0.559 | -1.454 | 0.158 | 0.284 |
| Pericalcarine Cortex | -1.274 | 0.227 | 0.423 | -0.562 | 0.584 | 0.751 | -1.05 | 0.304 | 0.405 |
| Pars Triangularis | -0.534 | 0.603 | 0.678 | 0.258 | 0.800 | 0.883 | -0.494 | 0.626 | 0.663 |
| Isthmus of the Cingulate Gyrus | -1.334 | 0.207 | 0.423 | -0.967 | 0.351 | 0.559 | -1.599 | 0.122 | 0.264 |
| Pars Opercularis | 0.068 | 0.947 | 0.950 | -0.016 | 0.988 | 0.988 | -0.684 | 0.500 | 0.563 |
| Entorhinal Cortex | -1.084 | 0.300 | 0.456 | -2.331 | **0.0365** | 0.386 | -2.476 | **0.0201** | 0.181 |
| Rostral Middle Frontal Cortex | -1.057 | 0.312 | 0.456 | -0.291 | 0.776 | 0.883 | -0.573 | 0.572 | 0.624 |
| Superior Frontal Cortex | 0.064 | 0.950 | 0.950 | 0.080 | 0.938 | 0.965 | -0.823 | 0.418 | 0.519 |
| Frontal Pole | -2.384 | **0.0345** | 0.317 | -1.485 | 0.161 | 0.439 | -1.887 | 0.07 | 0.264 |
| Supramarginal Cortex | -0.933 | 0.359 | 0.484 | -0.473 | 0.644 | 0.799 | -0.745 | 0.463 | 0.538 |
| Banks of the Superior Temporal Sulcus | -1.735 | 0.108 | 0.324 | -1.394 | 0.187 | 0.439 | -1.267 | 0.217 | 0.340 |
| Temporal Pole | -1.939 | 0.0764 | 0.317 | -1.452 | 0.170 | 0.439 | -1.1 | 0.282 | 0.405 |
| Caudal Middle Frontal Cortex | 0.213 | 0.835 | 0.884 | 0.247 | 0.809 | 0.883 | 0.12 | 0.905 | 0.905 |
| Middle Temporal Cortex | -1.665 | 0.122 | 0.324 | -0.912 | 0.378 | 0.567 | -0.328 | 0.745 | 0.766 |
| Inferior Temporal Cortex | -2.242 | **0.0446** | 0.317 | -1.659 | 0.121 | 0.439 | -1.355 | 0.187 | 0.308 |
| Precuneus | -2.558 | **0.0251** | 0.317 | -1.010 | 0.331 | 0.559 | -1.729 | 0.096 | 0.264 |
| Para Hippocampal Gyrus | -0.574 | 0.577 | 0.670 | -0.412 | 0.687 | 0.824 | -1.577 | 0.127 | 0.264 |
| Cuneus | -1.912 | 0.080 | 0.317 | -1.029 | 0.322 | 0.559 | -1.579 | 0.126 | 0.264 |
| Fusiform Gyrus | -2.014 | 0.067 | 0.317 | -1.426 | 0.177 | 0.439 | -1.76 | 0.0902 | 0.264 |
| Inferior Parietal Cortex | -2.105 | 0.057 | 0.317 | -1.123 | 0.282 | 0.559 | -0.764 | 0.452 | 0.538 |
| Superior Parietal Cortex | -1.706 | 0.114 | 0.324 | -1.100 | 0.291 | 0.559 | -1.234 | 0.228 | 0.342 |
| Lateral Occipital Cortex | -2.231 | **0.0455** | 0.317 | -1.367 | 0.195 | 0.439 | -1.649 | 0.111 | 0.264 |
| Lingual Cortex | -2.089 | 0.0587 | 0.317 | -1.763 | 0.101 | 0.439 | -1.517 | 0.141 | 0.267 |
